# Serum total bile acid levels assist in the prediction of acute intussusception with abdominal type Henoch-Schonlein purpura in children

**DOI:** 10.3389/fped.2023.1183470

**Published:** 2023-06-05

**Authors:** Sijie Yu, Wei Feng, Yi Wang, Maoyuan Zhao, Yuying Tu, Zhenhua Guo

**Affiliations:** ^1^ Department of Nephrology, Children’s Hospital of Chongqing Medical University, Chongqing, China; ^2^ Ministry of Education Key Laboratory of Child Development and Disorders, Children's Hospital of Chongqing Medical University, Chongqing, China; ^3^ National Clinical Research Center for Child Health and Disorders (Chongqing), Children's Hospital of Chongqing Medical University, Chongqing, China; ^4^ China International Science and Technology Cooperation Base of Child Development and Critical Disorders, Children's Hospital of Chongqing Medical University, Chongqing, China; ^5^ Chongqing Key Laboratory of Pediatrics, Children's Hospital of Chongqing Medical University, Chongqing, China; ^6^ Department of General Surgery, Children’s Hospital of Chongqing Medical University, Chongqing, China; ^7^ Department of General Surgery, Jiangxi Hospital Affiliated to Children’s Hospital of Chongqing Medical University, Jiangxi, China; ^8^ Jiangxi Children’s Medical Center, Jiangxi Maternal and Child Health Hospital, Jiangxi, China

**Keywords:** total bile acid (TBA), acute intussusception, Henoch-Schönlein purpura, children, prediction

## Abstract

**Background:**

The severe acute abdomen associated with Henoch-Schonlein purpura (HSP) is an acute intussusception (AI). There is no reliable specific marker for AI with abdominal-type HSP. The serum total bile acid (TBA) level is a new prognostic marker associated with the severity of intestinal inflammation. The purpose of this study was to identify the prognostic value of serum TBA levels for the diagnosis of AI in children with abdominal-type HSP.

**Methods:**

A retrospective study of 708 patients with abdominal-type HSP was conducted, with demographic data, clinical symptoms, hepatic function index, immune function markers, and clinical outcomes assessed. Patients were divided into two groups: HSP (613 patients) and HSP with AI (95 patients). The data were analysed using SPSS 22.0.

**Results:**

Of the 708 patients, the serum TBA levels were higher in the HSP with AI group than in the HSP group (*P* < 0.05). Logistic regression analysis showed that vomiting (OR = 396.492, 95% CI = 14.93–10,529.67, *P* < 0.001), haematochezia (OR = 87.436, 95% CI = 5.944–1,286.214, *P* = 0.001), TBA (OR = 16.287, 95% CI = 4.83–54.922, *P* < 0.001), and D-dimer (OR = 5.987, 95% CI = 1.892–15.834, *P* = 0.003) were independent risk factors for abdominal-type HSP with AI. Receiver operating characteristic (ROC) curve analysis showed that the optimal cut-off serum TBA value (sensitivity = 91.58%, specificity = 84.67%, AUC = 93.6524%) was >3 μmol/L for predicting AI in children with abdominal-type HSP. In this group of HSP patients with AI, a serum TBA level ≥6.98 μmol/L was significantly associated with an increased incidence of operative treatment (51.85% vs. 75.61%, *P* = 0.0181), intestinal necrosis (9.26% vs. 29.27%, *P* = 0.0117), and length of hospital stay [15.76 ± 5.31 vs. 10.98 ± 2.83 (days), *P* < 0.0001].

**Conclusion:**

In children with HSP and AI, the serum TBA level was significantly higher. A novel but promising haematological indicator, the serum TBA level, helps identify HSP with and without AI and predicts intestinal necrosis in HSP with AI.

## 1. Introduction

Henoch-Schonlein purpura (HSP) is an immunological disorder characterized by inflammatory lesions of tiny arteries throughout the body and is more frequent in children (1). It manifests clinically as skin purpura, gastrointestinal problems, and joint discomfort. Involvement of the gastrointestinal tract occurs in 50%–75% of cases. The most common symptoms are colicky abdominal discomfort, vomiting, and gastrointestinal haemorrhage (2). A total of 1%–5% of patients have been reported to suffer from acute intussusception (AI) (3). AI is an intestinal obstruction caused by the insertion of the intestine and its connected mesentery into the adjacent intestinal lumen, characterized by an abrupt onset, rapid progression, and severe disease (4). If patients do not receive prompt and successful treatment, it may result in intestinal blood circulation disruption, which may eventually lead to intestinal necrosis, peritonitis, intestinal perforation, and other consequences; therefore, early diagnosis and therapy are required to ensure treatment effectiveness.

AI is the most common acute abdomen associated with HSP (5). However, the number of instances documented in the previous literature is modest, and the clinical attention is insufficient. The digestive system symptoms of abdominal HSP are primarily abdominal pain, vomiting, bloody stools, and intestinal enlargement as a result of oedema and bleeding, and a mass might be felt on physical examination (6). Therefore, if there are no pathognomonic symptoms, it might be challenging to make the diagnosis of HSP complicated with AI (7). This leads to a misdiagnosis and a missed AI diagnosis, which delays the diagnosis and treatment of the disease. There are currently no efficient or sensitive indications for predicting the occurrence of AI in children with HSP. Therefore, a thorough assessment of the associated risk factors is needed.

Bile acids (BAs) play an important role in the body's endogenous metabolites. BAs in the intestine are involved in lipid absorption and the regulation of intestinal motility, mucosal secretory function, and intestinal barrier function (8). BAs are involved in the progression of inflammatory bowel disease (9, 10) and irritable bowel syndrome (11) by interacting with the gut flora. A recent study indicated that abdominal-type HSP is associated with significant compositional and structural changes in the gut microbiota (12). The damage to the epithelium in the midgut of children with HSP changes the intestinal environment on which the bacteria depends on for survival, and the symbiotic bacteria's protective mechanism is destroyed (12). Intestinal mucosal epithelial damage in children with abdominal HSP is particularly serious, resulting in the rejection of the normal intestinal symbiotic bacteria and an imbalance of the intestinal flora (13). However, the utility of serum total bile acid (TBA) in predicting AI in children with abdominal-type HSP is largely unknown.

In this study, we assessed whether there was a connection between the level of serum TBA and the occurrence of AI in patients with abdominal type HSP.

## 2. Materials and methods

### 2.1. Patients

The data of children with abdominal-type HSP admitted to the Nephrology Department and General Surgery Department of Children's Hospital of Chongqing Medical University between January 2017 and December 2021 were retrospectively reviewed. All abdominal-type HSP patients fulfilled the diagnostic standards that were jointly established in 2010 by the Paediatric Rheumatology International Trials Organization and the European League Against Rheumatism (14). The inclusion criteria were (1) age from 1 to 14 years old and (2) diagnosis of HSP and gastrointestinal symptoms such as abdominal pain, vomiting, and haematochezia. The exclusion criteria were (1) incomplete clinical data; (2) patients with diseases of the liver, kidneys, heart, brain, and haematopoietic system; (3) severe infection; and (4) association with other types of rheumatic immune diseases. Data from 1,017 patients were initially retrieved, and all of them were confirmed to have abdominal-type HSP by the inclusion criteria. In addition, 309 patients were excluded, permitting 708 subjects for the following study.

### 2.2. Study design

The subject characteristics included (1) demographic data such as sex, age, first episode, and onset seasons and (2) clinical features such as gastrointestinal symptoms (abdominal pain, vomiting, haematemesis, haematochezia) and whether receiving glucocorticoid therapy within 72 h of the onset of gastrointestinal symptoms.

The hepatic function index (serum total bilirubin, serum direct bilirubin, gamma-glutamyltransferase, albumin), coagulation function parameters (thrombin time, prothrombin time, activated partial thromboplastin time, fibrinogen, D-dimer), and immune function markers (C3, C4, IgG, IgE, IgA, IgM) were collected. When gastrointestinal symptoms were present, the serum TBA level was measured.

### 2.3. Statistics

For data entry, Excel software was used, and statistical analyses were carried out with SPSS version 22.0 (IBM corporation, Armonk, NY, United States). *P* < 0.05 was regarded as statistically significant. The Shapiro Wilk test was used to determine whether the data were normally distributed. Values with a normal distribution are represented by the mean and standard deviation (mean ± SD), whereas values without a normal distribution are represented by medians and interquartile ranges. Numbers and percentages are used to represent categorical variables. The area under the curve and the cut-off value for the best predictor were determined using receiver operating characteristic (ROC) curve analysis (AUC). The mean and 95% confidence interval (95% CI) of continuous variables were compared using Student's *t* test. Chi-square or Fisher exact tests were used to compare categorical variables between the groups. Univariate and multivariate logistic regressions were also used to calculate odds ratios (ORs), 95% confidence intervals (CIs), and *P* values.

## 3. Results

### 3.1. Patient characteristics

**Figure 1:**
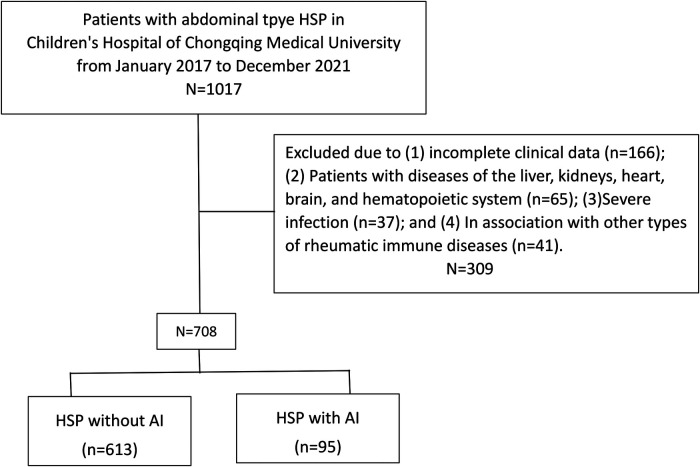
Population flowchart for the research project.

In the end, 708 patients with abdominal-type HSP involvement were included in this study (Figure 1). Table 1 summarizes the general characteristics of the study subjects with and without AI at baseline. The 613 children in the HSP without AI group included 357 (58.24%) boys and 256 (41.76%) girls. The ages ranged from 2 years and 2 months to 13 years and 9 months, with a median of 5 years and 8 months. Six hundred and one (98.04%) patients were going through their first HSP episode. The 95 children in the HSP with AI group included 56 (58.95%) boys and 39 (41.05%) girls. The ages ranged from 2 years and 1 month to 13 years and 2 months, with a median of 6 years and 5 months. Ninety-two (96.84%) patients were experiencing their first HSP episode. The onset season was primarily autumn and winter, with 16.67% (118/708) of the patients having episodes that occurred in the spring, 14.97% (106/708) of patients having them in the summer, 38.28% (271/708) of patients having them in the autumn, and 30.08% (213/708) of patients having them in the winter.

**Table 1:** General baseline characters of the study patients between study groups with and without acute intussusception in this study.

	HSP with AI (*n* = 95)	HSP without AI (*n* = 613)	*χ* ^2^	*P* value
Gender, *n* (%)			0.01	0.9118
Male	56 (58.95%)	357 (58.24%)		
Female	39 (41.05%)	256 (41.76%)		
Age, *n* (%)			5.43	**0** **.** **0198**
≥7 years	53 (55.79%)	438 (71.45%)		
<7 years	42 (44.21%)	175 (28.55%)		
First episode, *n* (%)			0.03	0.8697
Yes	92 (96.84%)	601 (98.04%)		
No	3 (3.16%)	12 (1.96%)		
Onset seasons, *n* (%)			7.01	0.0717
Spring	23 (24.21%)	95 (15.50%)		
Summer	8 (8.42%)	98 (15.99%)		
Autumn	37 (38.95%)	234 (38.17%)		
Winter	27 (28.42%)	186 (30.34%)		
Abdominal pain, *n* (%)			0.24	0.6229
Yes	95 (100%)	591 (96.41%)		
No	0	22 (3.59%)		
Vomiting, *n* (%)			196.85	**<0** **.** **0001**
Yes	84 (88.42%)	112 (18.27%)		
No	11 (11.58%)	501 (81.73%)		
Hematemesis, *n* (%)			0.09	0.7635
Yes	4 (4.21%)	22 (3.59%)		
No	91 (95.79%)	591 (96.41%)		
Hematochezia, *n* (%)			204.11	**<0** **.** **0001**
Yes	56 (58.95%)	37 (6.04%)		
No	39 (41.05%)	576 (93.96%)		
Receiving glucocorticoid therapy within 72 h of GI symptom emergence, *n* (%)			12.09	**0** **.** **0005**
Yes	52 (54.74%)	482 (78.63%)		
No	43 (45.26%)	131 (21.37%)		
Involved kidneys, *n* (%)			0.14	0.7085
Yes	303 (49.43%)	45 (47.37%)		
No	310 (50.57%)	50 (52.63%)		
White blood cell count, *n* (%)			0.23	0.6347
<10.0 × 10^9^/L	39 (41.05%)	236 (38.50%)		
≥10.0 × 10^9^/L	56 (58.95%)	377 (61.50%)		
Hemoglobin, *n* (%)			0.18	0.6752
<120 g/L	28 (29.47%)	168 (27.41%)		
≥120 g/L	67 (70.53%)	445 (72.59%)		
Neutrophil count, *n* (%)			0.98	0.3219
<7.5 × 10^9^/L	298 (48.61%)	41 (43.16%)		
≥7.5 × 10^9^/L	315 (51.39%)	54 (56.84%)		
Platelet count, *n* (%)			3.25	0.0712
<300.0 × 10^9^/L	18 (18.95%)	170 (27.73%)		
≥300.0 × 10^9^/L	77 (81.05%)	443 (72.27%)		
CRP, *n* (%)			0.74	0.0531
<8 mg/L	59 (62.11%)	447 (72.92%)		
≥8 mg/L	36 (37.89%)	166 (27.08%)		

GI, gastrointestinal symptom; AI, acute intussusception; CRP, C-reactive protein. The presence of bold values indicates that the difference between the two groups for this variable was statistically significant (*P* < 0.05).

Gender, first disease episode, and onset seasons had no statistically significant between-group differences (*P* > 0.05). There were statistically significant differences between the groups in age at onset (*P* = 0.0198) and whether glucocorticoid therapy was started within 72 h of the onset of gastrointestinal symptoms (*P* = 0.0005). In terms of symptoms, there were statistically significant differences in vomiting and haematochezia rates between the two groups (*P* < 0.001) but not in haematemesis rates and abdominal pain (*P* > 0.05). The involvement of the kidneys was also not significantly different (*P* > 0.05). Blood analysis revealed no statistically significant difference in white blood cell count, haemoglobin, neutrophil count, platelet count, or C-reactive protein levels between the study groups (*P* > 0.05).

All 95 AI patients had abdominal colour Doppler ultrasonography, which revealed “concentric circles” and/or the “false kidney sign.” Colour Doppler ultrasonography and x-rays were used to evaluate the children who had an intussusception air enema. Following the diagnosis of AI, the children received appropriate treatment evaluation of their overall physical wellbeing. Due to the long onset time or total peritonitis, 13 patients were operated on directly, and 82 patients were in fair condition with no obvious shock or perforation. According to imaging manifestations, 45 cases of ileocolic intussusceptions and 37 cases of small intestine intussusceptions had been identified. Air enema was performed with x-ray monitoring, and 31 patients (68.89%) had successful procedures in ileocolic intussusceptions. The success rate of air enema for small intestine intussusception was only 13.51% (5/37). Air enema failed in 46 patients (56.10%) following surgery. Of the 59 patients who underwent surgery, 17 (28.81%) underwent intestinal resection due to intestinal necrosis. There were 9 cases of ileal intussusception, 7 cases of ileocolic intussusception, and 1 case of jejunal intussusception among the 17 cases of enterectomy. The average nested distance in intussusception was 29 ± 15 cm. There were 28 cases of ileocolic intussusception, 13 cases of ileal intussusception, and 1 case of ileal ileocolic intussusception among the 42 children who underwent manual reduction, with an average nested distance in intussusception of 13 ± 6 cm.

### 3.2. Serum TBA levels and other serum markers were linked to abdominal-type HSP with AI

The TBA level of the HSP with AI group was significantly higher than that of the HSP group (6.98 ± 3.38 vs. 1.91 ± 0.78, *P* < 0.0001), and there were no differences between the two groups in TBIL, DBIL, γ-GGT, and albumin (*P* > 0.05). Coagulation function tests indicated significant differences between the study groups in D-dimer (*P* = 0.0146) and FIB levels (*P* = 0.0103) but not in TT, PT, or APTT (*P* > 0.05). Humoral immunity tests showed a statistically significant difference in serum total IgG (<0.0001) and IgM (*P* = 0.0002) between the study groups but not in IgE and IgA (*P* > 0.05; Table 2). There were no differences between the two groups in complement C3 and C4.

**Table 2:** Comparison of serum markers between the two groups.

	HSP with AI (*n* = 95)	HSP without AI (*n* = 613)	*t*/*χ*^2^	*P* value
TBIL (μmol/L), mean ± SD	8.39 ± 4.17	8.58 ± 4.20	0.39	0.6989
DBIL (μmol/L), mean ± SD	1.65 ± 1.73	2.08 ± 1.48		
γ-GGT (IU/L), mean ± SD	11.59 ± 4.30	13.53 ± 3.77	1.51	0.1308
Albumin (g/L), *n* (%)			1.84	0.1744
<30 g/L	11 (11.58%)	46 (7.50%)		
≥30 g/L	84 (88.42%)	567 (92.50%)		
TBA (umol/L), mean ± SD	6.98 ± 3.38	1.91 ± 0.78	29.99	**<0** **.** **0001**
D-dimer (mg/L), mean ± SD	3.96 ± 1.83	10.63 ± 3.57	12.00	**<0** **.** **0001**
TT(s), mean ± SD	16.75 ± 1.49	16.48 ± 0.99	1.67	0.0957
PT(s), mean ± SD	11.72 ± 0.62	11.84 ± 1.41	0.55	0.5848
APTT(s), mean ± SD	27.36 ± 4.04	27.58 ± 2.17	0.12	0.933
FIB (g/L), mean ± SD	2.77 ± 0.69	3.02 ± 0.23	2.57	**0** **.** **0103**
Complement C3 (g/L), mean ± SD	0.95 ± 0.18	0.98 ± 0.12	1.20	0.2296
Complement C4 (g/L), mean ± SD	0.21 ± 0.04	0.22 ± 0.23	1.36	0.1732
IgG (g/L), mean ± SD	7.69 ± 2.66	9.06 ± 3.46	4.19	**<0** **.** **0001**
IgE (IU/mL), mean ± SD	94.96 ± 31.97	169.30 ± 76.95	1.33	0.1842
IgA (g/L), mean ± SD	1.90 ± 0.54	2.03 ± 0.23	1.35	0.1781
IgM (g/L), mean ± SD	0.97 ± 1.13	1.20 ± 0.19	3.70	**0** **.** **0002**

TBIL, serum total bilirubin; DBIL, serum direct bilirubin; γ-GGT, gamma-glutamyltransferase; TBA, serum total bile acid; TT, thrombin time; PT, prothrombin time; APTT, activated partial thromboplastin time; FIB, fibrinogen. The presence of bold values indicates that the difference between the two groups for this variable was statistically significant (*P* < 0.05).

### 3.3. Logistic regression analysis of potential risk factors for AI in children with abdominal-type HSP

The logistic regression equation included ten factors with *P* < 0.05 in the univariate analysis. The risk factors for abdominal-type HSP with AI were investigated using stepwise multiple regression analysis. The results showed that vomiting (OR = 396.492, 95% CI = 14.93–10,529.67, *P* < 0.001), haematochezia (OR = 87.436, 95% CI = 5.944–1,286.214, *P* = 0.001), TBA (OR = 16.287, 95% CI = 4.83–54.922, *P* < 0.001), and D-dimer (OR = 5.987, 95% CI = 1.892–15.834, *P* = 0.003) were independent risk factors for abdominal-type HSP with AI (Table 3).

**Table 3:** Independent influencing factors for abdominal type HSP with AI.

Characteristics	*β*	SE	OR	95% CI	*Z*	*P*
Age	0.514	0.40857	1.673	0.751–3.726	1.259	0.208
Vomiting	5.983	1.67311	396.492	14.93–10,529.67	3.576	**<0** **.** **001**
Hematochezia	4.471	1.37171	87.436	5.944–1,286.214	3.259	**0** **.** **001**
Glucocorticoid therapy	−1.857	1.10876	0.156	0.018–1.372	−1.675	0.094
TBA	2.79	0.62017	16.287	4.83–54.922	4.499	**<0** **.** **001**
D-dimer	1.546	0.6758	5.987	1.892–15.834	3.475	**0** **.** **003**
FIB	−1.438	0.64415	0.237	0.067–0.839	1.128	0.259
IgG	−0.037	0.06709	0.964	0.845–1.099	−0.551	0.582
IgM	−0.442	0.32858	0.643	0.338–1.224	−1.345	0.179

β, regression coefficient; SE, standard error; OR, odds ratio; 95% CI, 95% confidence interval; WBC, white blood cell count; TBA, serum total bile acid; FIB, fibrinogen. In multivariate Logistic regression analysis, the presence of bold values indicates that the difference between the two groups for this variable was statistically significant (*P* < 0.05).

### 3.4. Diagnostic utility of serum TBA for abdominal-type HSP with AI

**Figure 2:**
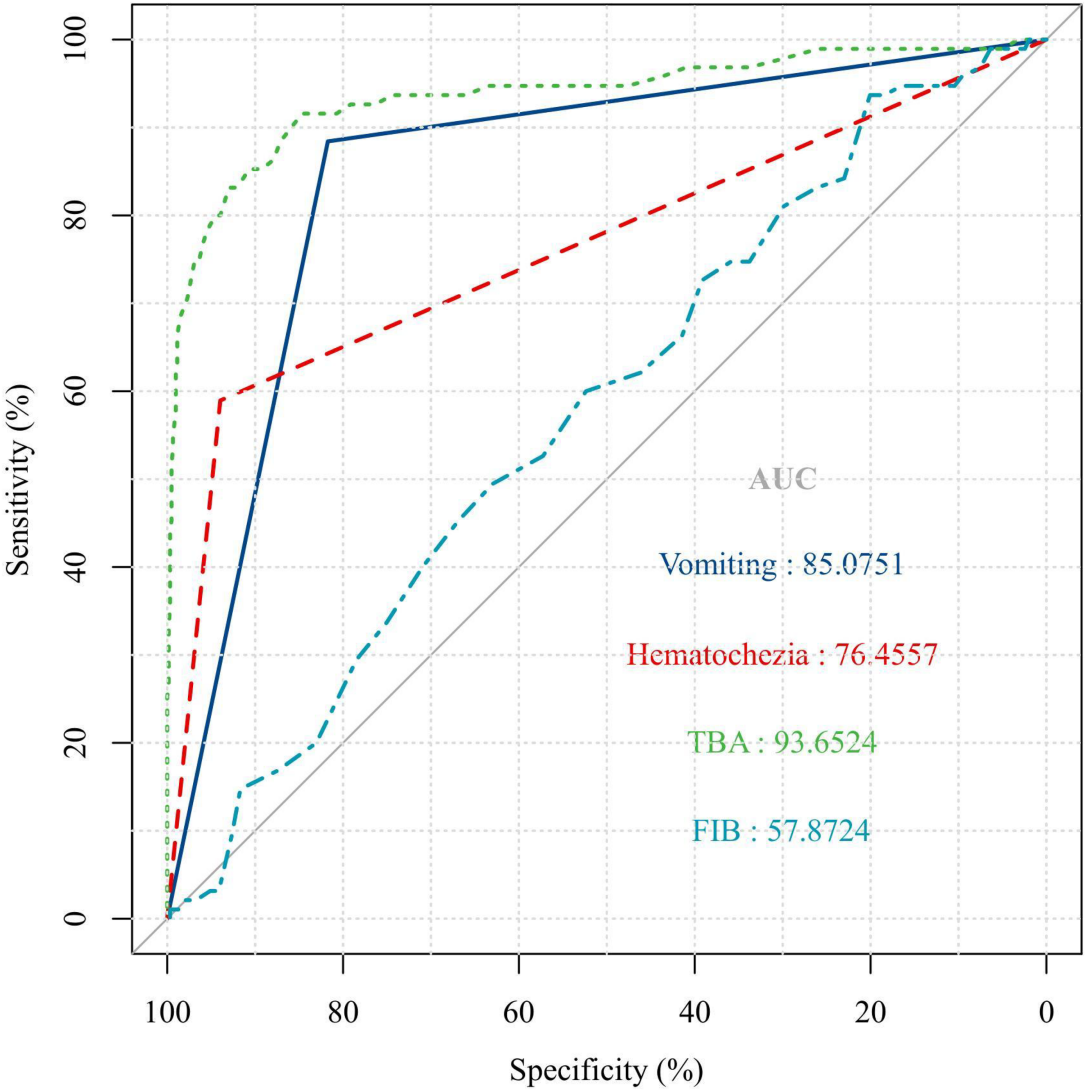
ROC curve analysis. The optimal serum TBA value cut off of >3 μmol/L for predicting AI in children with abdominal type HSP (sensitivity = 91.58%, specificity = 84.67%, *P* < 0.001).

The ROC curve analysis showed that the optimal serum TBA value cut-off was >3 μmol/L for predicting AI in children with abdominal-type HSP (sensitivity = 91.58%, specificity = 84.67%, AUC = 93.6524%). The ROC analysis revealed that a serum D-dimer cut-off level of 4.7 mg/L provides the prediction for abdominal-type HSP with AI in this cohort, with a sensitivity of 93.68% but a specificity of only 20.07% (AUC = 57.8724%) (Figure 2).

In this cohort, vomiting (sensitivity = 88.42%, specificity = 81.73%, AUC = 85.0751%) and haematochezia (sensitivity = 58.95%, specificity = 93.96%, AUC = 76.4557) were the best clinical predictors for abdominal-type HSP with AI (Figure 2).

### 3.5. Outcomes based on HSP with AI and serum TBA level

In this group of HSP patients with AI, the average serum TBA level was 6.98 μmol/L. According to the average level of TBA, the group comparison showed that a TBA level ≥6.98 μmol/L was significantly associated with an increased incidence of operative treatment (51.85% vs. 75.61%, *P* = 0.0181), intestinal necrosis (9.26% vs. 29.27%, *P* = 0.0117), and length of hospital stay [15.76 ± 5.31 vs. 10.98 ± 2.83 (days), *P* < 0.0001] (Table 4).

**Table 4:** Outcomes based on the HSP with AI and serum TBA level.

Variables	TBA < 6.98 μmol/L (*n* = 54)	TBA ≥ 6.98 μmol/L (*n* = 41)	*t*/*χ*^2^	*P* value
Operative technique^a^	28 (51.85%)	31 (75.61%)	5.59	**0** **.** **0181**
Intestinal necrosis, *n* (%)^b^	4 (7.41%)	13 (31.71%)	9.37	**0** **.** **0022**
LOS (days)^c^	10.98 ± 2.83	15.76 ± 5.31	4.21	**<0** **.** **0001**

The presence of bold values indicates that the difference between the two groups for this variable was statistically significant (*P* < 0.05).

^a^ The air enema was unsuccessful. Chi-square test.

^b^ Direct chi-square test.

^c^ LOS, length of hospital stay. *t*-test.

## 4. Discussion

The current study found that serum TBA levels, fibrinogen, vomiting, and haematochezia were independently associated with abdominal-type HSP and AI. In terms of prediction, TBA had a sensitivity of 91.58% and a specificity of 84.67% in identifying children at high risk for AI. In addition, an average serum TBA level of 6.98 mol/L was significantly associated with an increased incidence of surgery, intestinal necrosis, and length of hospital stay in HSP patients with AI.

Children with HSP experience widespread intestinal wall oedema and segmental bleeding of the subserous membrane and mucosa as a result of antigens and antibodies acting on intestinal wall capillaries, increasing capillary permeability (15). Intussusception occurs when the lateral intestine is pushed into the distal bowel and the intestinal segment with the stiff intestinal wall is drawn near because of oedema and bleeding (16). Early diagnosis of intussusception is dependent on the clinical presentation of the patient and timely ultrasound examination. The gastrointestinal symptoms of abdominal-type HSP are frequently similar to intussusception, resulting in misdiagnosis and disease delay. When the diagnosis of intussusception was confirmed within 4 h of being admitted to a hospital, the success rate of an enema was markedly increased, and the rate of intestinal resection was lower, which emphasizes the significance of an early and prompt diagnosis (17). Furthermore, the current study found that vomiting and haematochezia were independent risk factors for the development of intussusception in children with abdominal-type HSP. Gastrointestinal bleeding is more common in paediatric HSP cases with gastrointestinal (GI) engagement and intestinal perforation (18). Intussusception has a pathological basis in intestinal obstruction, and the most common presentation has been reported to be vomiting followed by rectal bleeding (19, 20). Vomiting and haematochezia should be considered as possible causes of acute surgical abdomen in paediatric HSP patients with GI involvement. HSP with intussusception, on the other hand, frequently lacks specific clinical manifestations. As a result, only a few children in the previously mentioned intussusception group displayed differential signs requiring surgical treatment, such as abdominal pain, rebound tenderness, and an abdominal mass. Ultrasound is recognized as a valuable diagnostic tool for the clinical diagnosis of intussusception (21).

Although the above variable is discussed in both previous studies and our study, it is important to note that those factors are greatly influenced by the attending physician's experience and are subjective. Furthermore, objective variables obtained from blood samples are usually more reproducible and thus more valuable. In a previous report, GI symptoms of HSP were correlated with serum biomarkers such as D-dimer, white blood cell count, and C-reactive protein level (7, 22). During the acute phase of HSP, D-dimer is more strongly related to disease activity and more consistently demonstrates GI involvement than inflammatory markers (22). D-dimer detection is important in the diagnosis of abdominal-type HSP, and D-dimer is one of the markers that specifically reflects the hypercoagulable state and secondary hyperfibrinolysis in the body (23). When abdominal HSP occurs, the gastrointestinal capillary endothelium is damaged, a large number of inflammatory factors are released, and exogenous coagulation pathways are activated, which can result in hypercoagulability and prethrombosis (24). When there is unexplained abdominal pain and an elevated D-dimer level, abdominal Henoch-Schonlein purpura should be suspected. Consistent with a previous report, our current research examined high D-dimer levels as an independent risk factor for intussusception in children with abdominal-type HSP, with a specificity of only 20.07%. Large-scale clinical trials are needed to determine whether D-dimer can be used as a prognostic marker in HSP with AI.

To diagnose HSP with intussusception in children, it is necessary to identify diagnostic noninvasive laboratory markers. Bile acids are important signalling molecules that regulate immunity as well as maintain and promote intestinal barrier function. Bile acids have been associated with colorectal cancer (25), irritable bowel syndrome (26), ulcerative colitis (27), and other diseases. The serum TBA levels were significantly higher in the intussusception group than in the HSP group in this study.

The TBA level at admission could guide the severity of HSP and predict prognosis, with 3 μmol/L being the best cut-off point. When TBA reached 3 μmol/L, the probability of intussusception complicated by HSP increased significantly.

Air enema and surgery are used to treat intussusception. The success rate of air enema is relatively low in children with HSP due to the serious secondary changes such as intestinal wall oedema and bleeding (28). The success rate of air enema in this study were only 68.89% and 13.51% in ileocolic intussusceptions and small intestine intussusceptions, respectively, and more than a quarter of patients underwent enterectomy due to intestinal necrosis or perforation. Because HSP is a self-limiting disease, it is feasible to identify it early and avoid unnecessary surgery. However, experienced clinicians are required to closely monitor the changes in patients’ conditions during the conservative treatment process. The most serious complication of conservative treatment for patients with intussusception is intestinal perforation. Manual and surgical treatment are required when there are signs of intestinal ischaemic necrosis and perforation. The surgical evidence is frequently difficult to grasp for these patients. When the average serum TBA in this group of intussusception patients was 6.98 µmol/L, the risk of operation and intestinal perforation was significantly increased, and the hospital stay was also prolonged. As a result, a high serum TBA level is an important predictor of a poor prognosis of intussusception.

There were several limitations to this study. This study used a retrospective case‒control design. In terms of research objects, a large number of HSP patients with incomplete data were excluded, which may have biased the findings. The subjects in this study had been hospitalized in the previous 5 years, and the descriptions of clinical indicators may be inconsistent. Although logistic regression analysis was used to some extent in the data analysis, the influence of clinical indicators remained significant, which may have interfered with the detection value between HSP with intussusception and serum TBA. In terms of assessing patient prognosis, the evaluation methods in this study are relatively one-sided, and there is still a lack of comprehensive evaluation data of intussusception injury, so the relationship between HSP with intussusception and serum TBA should be explored more comprehensively and accurately.

## 5. Conclusion

We demonstrated that serum TBA levels can assist clinicians to predict the occurrence of AI with abdominal type HSP in children. The serum TBA level can be used as a noninvasive and easily applicable biomarker for the screening and predicting progression in HSP with AI.

## Data Availability

The original contributions presented in the study are included in the article, further inquiries can be directed to the corresponding author.
